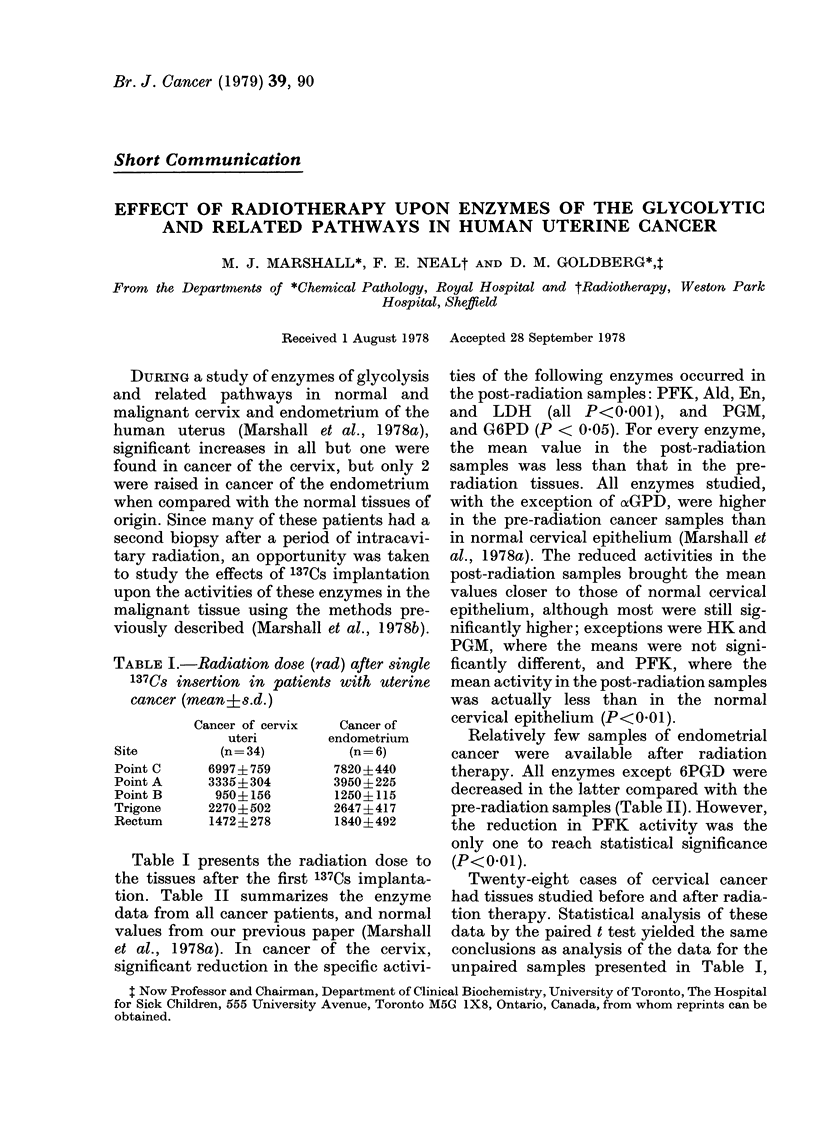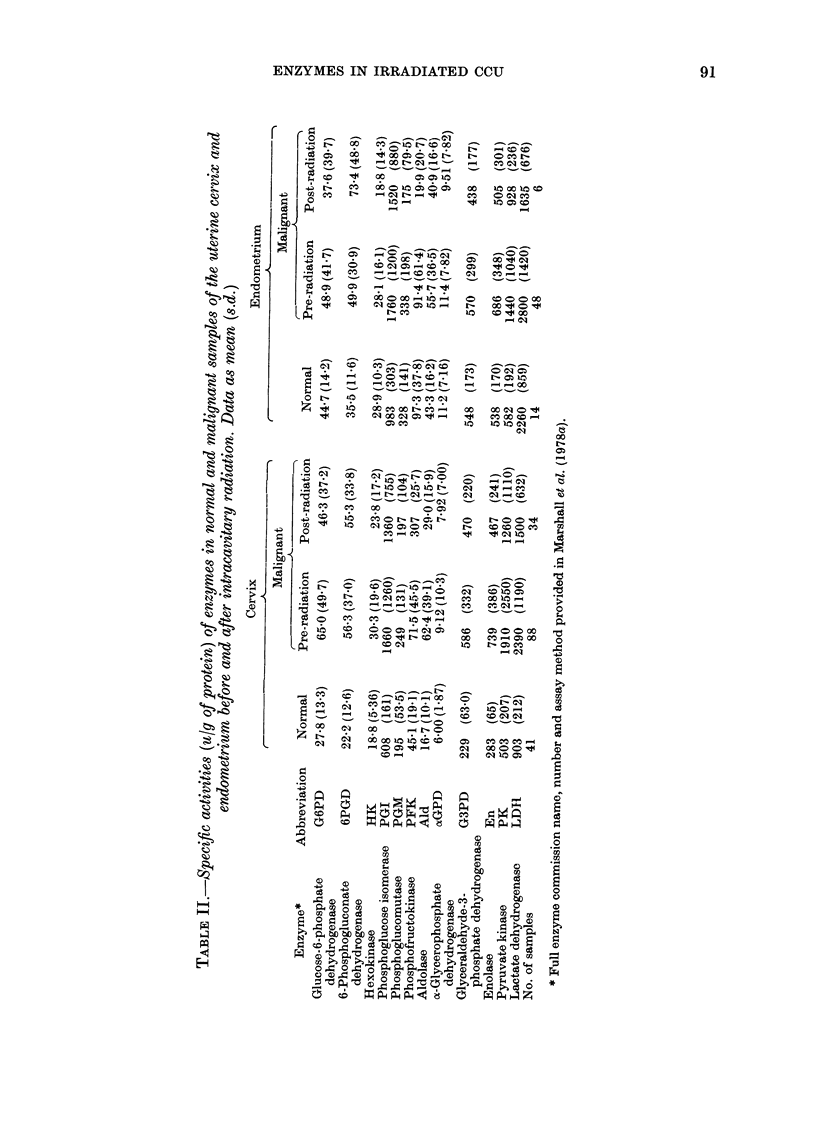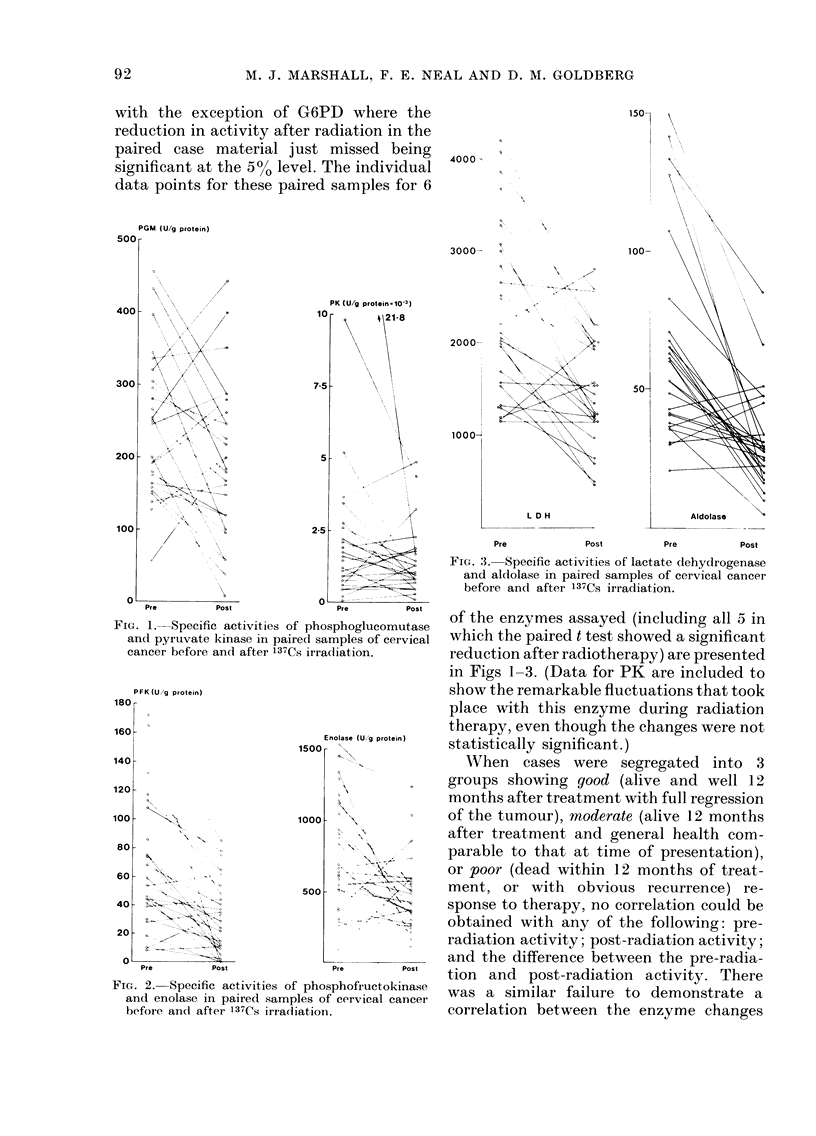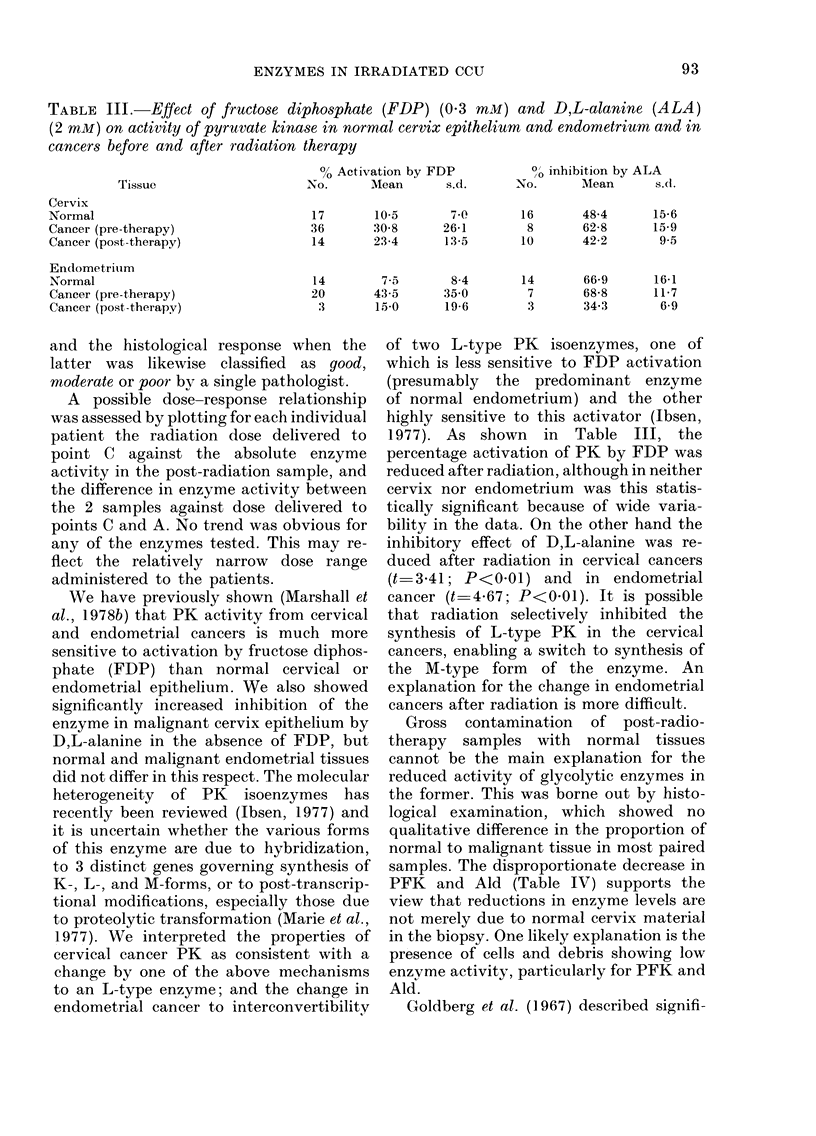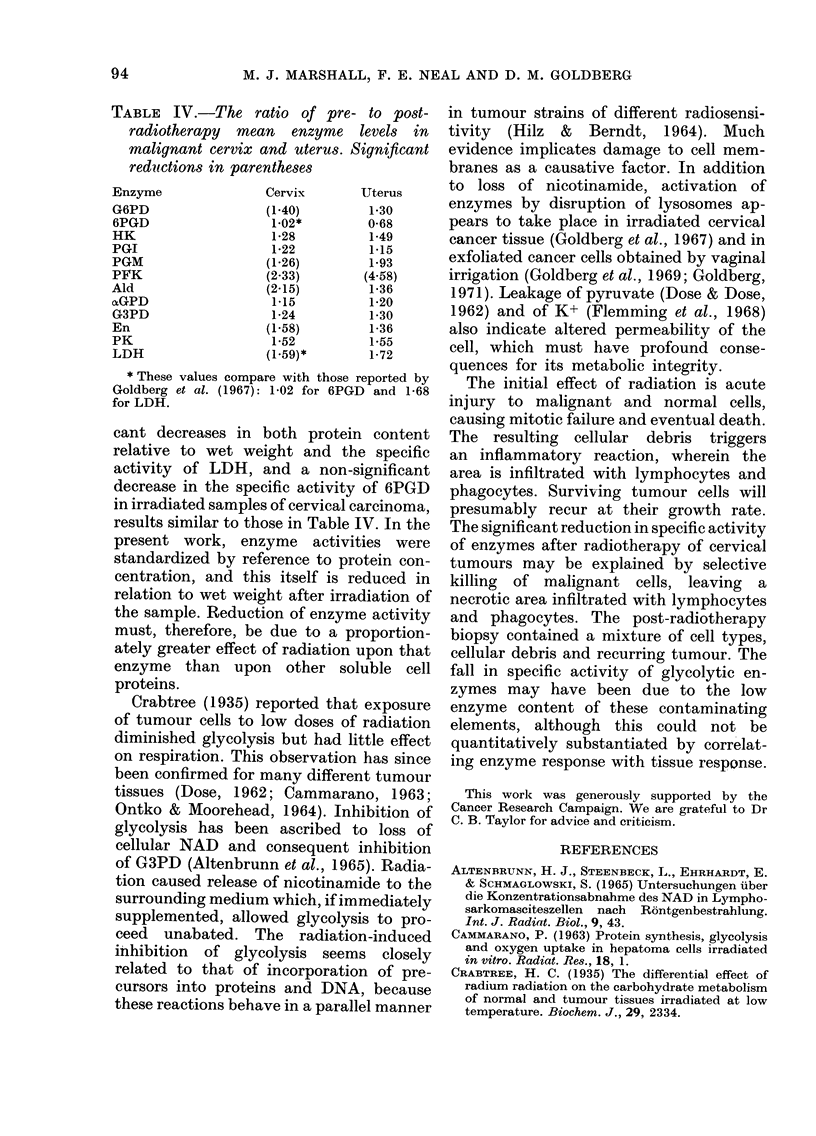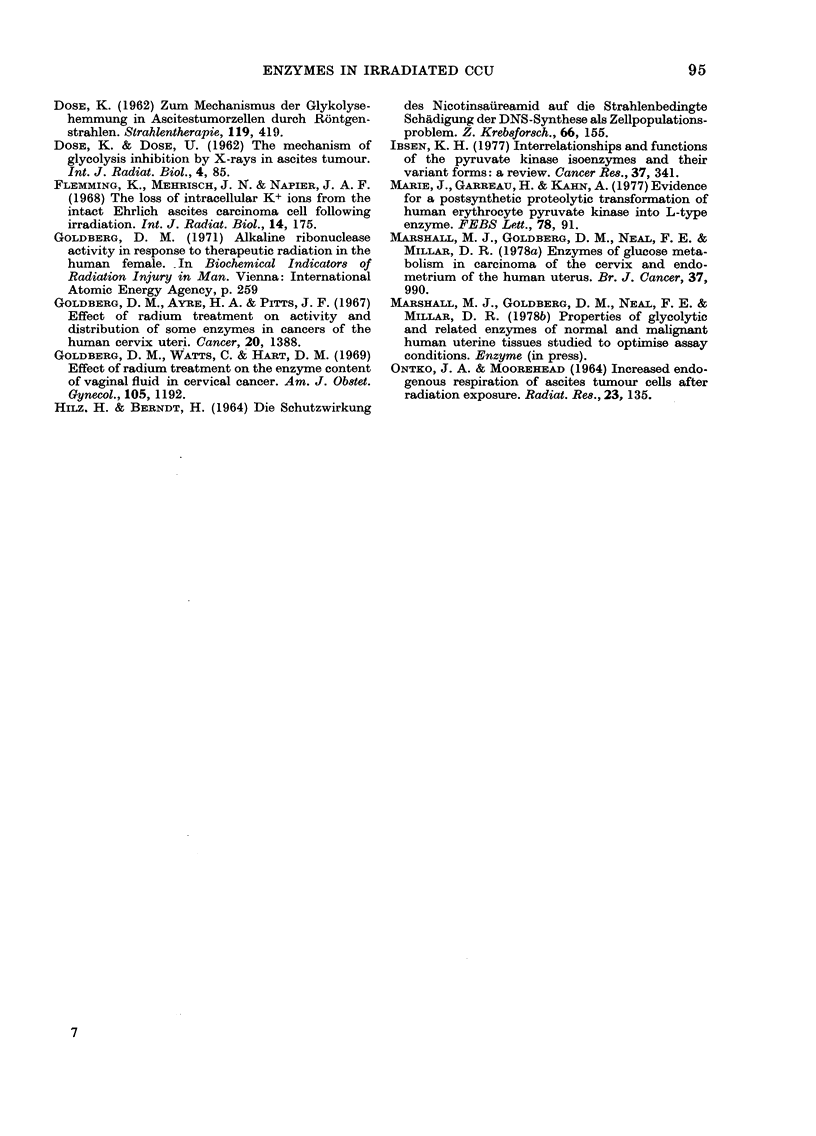# Effect of radiotherapy upon enzymes of the glycolytic and related pathways in human uterine cancer.

**DOI:** 10.1038/bjc.1979.11

**Published:** 1979-01

**Authors:** M. J. Marshall, F. E. Neal, D. M. Goldberg


*Br. J. Cancer* (1979) **39**, 90

***Short Communication***

### EFFECT OF RADIOTHERAPY UPON ENZYMES OF THE GLYCOLYTIC AND RELATED PATHWAYS IN HUMAN UTERINE CANCER

M. J. MARSHALL*, F. E. NEAL† AND D. M. GOLDBERG*,‡

*From the Departments of *Chemical Pathology, Royal Hospital and †Radiotherapy, Weston Park Hospital, Sheffield*

Received 1 August 1978 Accepted 28 September 1978

DURING a study of enzymes of glycolysis and related pathways in normal and malignant cervix and endometrium of the human uterus (Marshall *et al.*, 1978*a*), significant increases in all but one were found in cancer of the cervix, but only 2 were raised in cancer of the endometrium when compared with the normal tissues of origin. Since many of these patients had a second biopsy after a period of intracavitary radiation, an opportunity was taken to study the effects of $^{137}$Cs implantation upon the activities of these enzymes in the malignant tissue using the methods previously described (Marshall *et al.*, 1978*b*).

TABLE I.—*Radiation dose (rad) after single $^{137}$Cs insertion in patients with uterine cancer (mean±s.d.)*

| Site | Cancer of cervix uteri (n=34) | Cancer of endometrium (n=6) |
|---|---|---|
| Point C | 6997±759 | 7820±440 |
| Point A | 3335±304 | 3950±225 |
| Point B | 950±156 | 1250±115 |
| Trigone | 2270±502 | 2647±417 |
| Rectum | 1472±278 | 1840±492 |

Table I presents the radiation dose to the tissues after the first $^{137}$Cs implantation. Table II summarizes the enzyme data from all cancer patients, and normal values from our previous paper (Marshall *et al.*, 1978*a*). In cancer of the cervix, significant reduction in the specific activities of the following enzymes occurred in the post-radiation samples: PFK, Ald, En, and LDH (all $P<0.001$), and PGM, and G6PD ($P < 0.05$). For every enzyme, the mean value in the post-radiation samples was less than that in the pre-radiation tissues. All enzymes studied, with the exception of αGPD, were higher in the pre-radiation cancer samples than in normal cervical epithelium (Marshall *et al.*, 1978*a*). The reduced activities in the post-radiation samples brought the mean values closer to those of normal cervical epithelium, although most were still significantly higher; exceptions were HK and PGM, where the means were not significantly different, and PFK, where the mean activity in the post-radiation samples was actually less than in the normal cervical epithelium ($P<0.01$).

Relatively few samples of endometrial cancer were available after radiation therapy. All enzymes except 6PGD were decreased in the latter compared with the pre-radiation samples (Table II). However, the reduction in PFK activity was the only one to reach statistical significance ($P<0.01$).

Twenty-eight cases of cervical cancer had tissues studied before and after radiation therapy. Statistical analysis of these data by the paired *t* test yielded the same conclusions as analysis of the data for the unpaired samples presented in Table I,

‡ Now Professor and Chairman, Department of Clinical Biochemistry, University of Toronto, The Hospital for Sick Children, 555 University Avenue, Toronto M5G 1X8, Ontario, Canada, from whom reprints can be obtained.

TABLE II.—*Specific activities (u/g of protein) of enzymes in normal and malignant samples of the uterine cervix and endometrium before and after intracavitary radiation. Data as mean (s.d.)*

| | | Cervix | | | Endometrium | | |
|---|---|---|---|---|---|---|---|
| | | | Malignant | | | Malignant | |
| Enzyme* | Abbreviation | Normal | Pre-radiation | Post-radiation | Normal | Pre-radiation | Post-radiation |
| Glucose-6-phosphate dehydrogenase | G6PD | 27·8 (13·3) | 65·0 (49·7) | 46·3 (37·2) | 44·7 (14·2) | 48·9 (41·7) | 37·6 (39·7) |
| 6-Phosphogluconate dehydrogenase | 6PGD | 22·2 (12·6) | 56·3 (37·0) | 55·3 (33·8) | 35·5 (11·6) | 49·9 (30·9) | 73·4 (48·8) |
| Hexokinase | HK | 18·8 (5·36) | 30·3 (19·6) | 23·8 (17·2) | 28·9 (10·3) | 28·1 (16·1) | 18·8 (14·3) |
| Phosphoglucose isomerase | PGI | 608 (161) | 1660 (1260) | 1360 (755) | 983 (303) | 1760 (1200) | 1520 (880) |
| Phosphoglucomutase | PGM | 195 (53·5) | 249 (131) | 197 (104) | 328 (141) | 338 (198) | 175 (79·5) |
| Phosphofructokinase | PFK | 45·1 (19·1) | 71·5 (45·5) | 307 (25·7) | 97·3 (37·8) | 91·4 (61·4) | 19·9 (20·7) |
| Aldolase | Ald | 16·7 (10·1) | 62·4 (39·1) | 29·0 (15·9) | 43·3 (16·2) | 55·7 (36·5) | 40·9 (16·6) |
| α-Glycerophosphate dehydrogenase | αGPD | 6·00 (1·87) | 9·12 (10·3) | 7·92 (7·00) | 11·2 (7·16) | 11·4 (7·82) | 9·51 (7·82) |
| Glyceraldehyde-3-phosphate dehydrogenase | G3PD | 229 (63·0) | 586 (332) | 470 (220) | 548 (173) | 570 (299) | 438 (177) |
| Enolase | En | 283 (65) | 739 (386) | 467 (241) | 538 (170) | 686 (348) | 505 (301) |
| Pyruvate kinase | PK | 503 (207) | 1910 (2550) | 1260 (1110) | 582 (192) | 1440 (1040) | 928 (236) |
| Lactate dehydrogenase | LDH | 903 (212) | 2390 (1190) | 1500 (632) | 2260 (859) | 2800 (1420) | 1635 (676) |
| No. of samples | | 41 | 88 | 34 | 14 | 48 | 6 |

* Full enzyme commission name, number and assay method provided in Marshall *et al.* (1978*a*).

with the exception of G6PD where the reduction in activity after radiation in the paired case material just missed being significant at the 5% level. The individual data points for these paired samples for 6

FIG. 1.—Specific activities of phosphoglucomutase and pyruvate kinase in paired samples of cervical cancer before and after $^{137}Cs$ irradiation.

FIG. 2.—Specific activities of phosphofructokinase and enolase in paired samples of cervical cancer before and after $^{137}Cs$ irradiation.

FIG. 3.—Specific activities of lactate dehydrogenase and aldolase in paired samples of cervical cancer before and after $^{137}Cs$ irradiation.

of the enzymes assayed (including all 5 in which the paired *t* test showed a significant reduction after radiotherapy) are presented in Figs 1–3. (Data for PK are included to show the remarkable fluctuations that took place with this enzyme during radiation therapy, even though the changes were not statistically significant.)

When cases were segregated into 3 groups showing *good* (alive and well 12 months after treatment with full regression of the tumour), *moderate* (alive 12 months after treatment and general health comparable to that at time of presentation), or *poor* (dead within 12 months of treatment, or with obvious recurrence) response to therapy, no correlation could be obtained with any of the following: pre-radiation activity; post-radiation activity; and the difference between the pre-radiation and post-radiation activity. There was a similar failure to demonstrate a correlation between the enzyme changes

TABLE III.—*Effect of fructose diphosphate (FDP) (0·3 mM) and D,L-alanine (ALA) (2 mM) on activity of pyruvate kinase in normal cervix epithelium and endometrium and in cancers before and after radiation therapy*

| | % Activation by FDP | | | % inhibition by ALA | | |
|---|---|---|---|---|---|---|
| Tissue | No. | Mean | s.d. | No. | Mean | s.d. |
| Cervix | | | | | | |
| Normal | 17 | 10·5 | 7·0 | 16 | 48·4 | 15·6 |
| Cancer (pre-therapy) | 36 | 30·8 | 26·1 | 8 | 62·8 | 15·9 |
| Cancer (post-therapy) | 14 | 23·4 | 13·5 | 10 | 42·2 | 9·5 |
| Endometrium | | | | | | |
| Normal | 14 | 7·5 | 8·4 | 14 | 66·9 | 16·1 |
| Cancer (pre-therapy) | 20 | 43·5 | 35·0 | 7 | 68·8 | 11·7 |
| Cancer (post-therapy) | 3 | 15·0 | 19·6 | 3 | 34·3 | 6·9 |

and the histological response when the latter was likewise classified as *good*, *moderate* or *poor* by a single pathologist.

A possible dose–response relationship was assessed by plotting for each individual patient the radiation dose delivered to point C against the absolute enzyme activity in the post-radiation sample, and the difference in enzyme activity between the 2 samples against dose delivered to points C and A. No trend was obvious for any of the enzymes tested. This may reflect the relatively narrow dose range administered to the patients.

We have previously shown (Marshall *et al.*, 1978*b*) that PK activity from cervical and endometrial cancers is much more sensitive to activation by fructose diphosphate (FDP) than normal cervical or endometrial epithelium. We also showed significantly increased inhibition of the enzyme in malignant cervix epithelium by D,L-alanine in the absence of FDP, but normal and malignant endometrial tissues did not differ in this respect. The molecular heterogeneity of PK isoenzymes has recently been reviewed (Ibsen, 1977) and it is uncertain whether the various forms of this enzyme are due to hybridization, to 3 distinct genes governing synthesis of K-, L-, and M-forms, or to post-transcriptional modifications, especially those due to proteolytic transformation (Marie *et al.*, 1977). We interpreted the properties of cervical cancer PK as consistent with a change by one of the above mechanisms to an L-type enzyme; and the change in endometrial cancer to interconvertibility of two L-type PK isoenzymes, one of which is less sensitive to FDP activation (presumably the predominant enzyme of normal endometrium) and the other highly sensitive to this activator (Ibsen, 1977). As shown in Table III, the percentage activation of PK by FDP was reduced after radiation, although in neither cervix nor endometrium was this statistically significant because of wide variability in the data. On the other hand the inhibitory effect of D,L-alanine was reduced after radiation in cervical cancers ($t=3·41$; $P<0·01$) and in endometrial cancer ($t=4·67$; $P<0·01$). It is possible that radiation selectively inhibited the synthesis of L-type PK in the cervical cancers, enabling a switch to synthesis of the M-type form of the enzyme. An explanation for the change in endometrial cancers after radiation is more difficult.

Gross contamination of post-radiotherapy samples with normal tissues cannot be the main explanation for the reduced activity of glycolytic enzymes in the former. This was borne out by histological examination, which showed no qualitative difference in the proportion of normal to malignant tissue in most paired samples. The disproportionate decrease in PFK and Ald (Table IV) supports the view that reductions in enzyme levels are not merely due to normal cervix material in the biopsy. One likely explanation is the presence of cells and debris showing low enzyme activity, particularly for PFK and Ald.

Goldberg *et al.* (1967) described signifi-

TABLE IV.—*The ratio of pre- to post-radiotherapy mean enzyme levels in malignant cervix and uterus. Significant reductions in parentheses*

| Enzyme | Cervix | Uterus |
|---|---|---|
| G6PD | (1·40) | 1·30 |
| 6PGD | 1·02* | 0·68 |
| HK | 1·28 | 1·49 |
| PGI | 1·22 | 1·15 |
| PGM | (1·26) | 1·93 |
| PFK | (2·33) | (4·58) |
| Ald | (2·15) | 1·36 |
| αGPD | 1·15 | 1·20 |
| G3PD | 1·24 | 1·30 |
| En | (1·58) | 1·36 |
| PK | 1·52 | 1·55 |
| LDH | (1·59)* | 1·72 |

* These values compare with those reported by Goldberg *et al.* (1967): 1·02 for 6PGD and 1·68 for LDH.

cant decreases in both protein content relative to wet weight and the specific activity of LDH, and a non-significant decrease in the specific activity of 6PGD in irradiated samples of cervical carcinoma, results similar to those in Table IV. In the present work, enzyme activities were standardized by reference to protein concentration, and this itself is reduced in relation to wet weight after irradiation of the sample. Reduction of enzyme activity must, therefore, be due to a proportionately greater effect of radiation upon that enzyme than upon other soluble cell proteins.

Crabtree (1935) reported that exposure of tumour cells to low doses of radiation diminished glycolysis but had little effect on respiration. This observation has since been confirmed for many different tumour tissues (Dose, 1962; Cammarano, 1963; Ontko & Moorehead, 1964). Inhibition of glycolysis has been ascribed to loss of cellular NAD and consequent inhibition of G3PD (Altenbrunn *et al.*, 1965). Radiation caused release of nicotinamide to the surrounding medium which, if immediately supplemented, allowed glycolysis to proceed unabated. The radiation-induced inhibition of glycolysis seems closely related to that of incorporation of precursors into proteins and DNA, because these reactions behave in a parallel manner in tumour strains of different radiosensitivity (Hilz & Berndt, 1964). Much evidence implicates damage to cell membranes as a causative factor. In addition to loss of nicotinamide, activation of enzymes by disruption of lysosomes appears to take place in irradiated cervical cancer tissue (Goldberg *et al.*, 1967) and in exfoliated cancer cells obtained by vaginal irrigation (Goldberg *et al.*, 1969; Goldberg, 1971). Leakage of pyruvate (Dose & Dose, 1962) and of $K^+$ (Flemming *et al.*, 1968) also indicate altered permeability of the cell, which must have profound consequences for its metabolic integrity.

The initial effect of radiation is acute injury to malignant and normal cells, causing mitotic failure and eventual death. The resulting cellular debris triggers an inflammatory reaction, wherein the area is infiltrated with lymphocytes and phagocytes. Surviving tumour cells will presumably recur at their growth rate. The significant reduction in specific activity of enzymes after radiotherapy of cervical tumours may be explained by selective killing of malignant cells, leaving a necrotic area infiltrated with lymphocytes and phagocytes. The post-radiotherapy biopsy contained a mixture of cell types, cellular debris and recurring tumour. The fall in specific activity of glycolytic enzymes may have been due to the low enzyme content of these contaminating elements, although this could not be quantitatively substantiated by correlating enzyme response with tissue response.

This work was generously supported by the Cancer Research Campaign. We are grateful to Dr C. B. Taylor for advice and criticism.

### REFERENCES

ALTENBRUNN, H. J., STEENBECK, L., EHRHARDT, E. & SCHMAGLOWSKI, S. (1965) Untersuchungen über die Konzentrationsabnahme des NAD in Lymphosarkomasciteszellen nach Röntgenbestrahlung. *Int. J. Radiat. Biol.*, **9**, 43.

CAMMARANO, P. (1963) Protein synthesis, glycolysis and oxygen uptake in hepatoma cells irradiated *in vitro*. *Radiat. Res.*, **18**, 1.

CRABTREE, H. C. (1935) The differential effect of radium radiation on the carbohydrate metabolism of normal and tumour tissues irradiated at low temperature. *Biochem. J.*, **29**, 2334.

DOSE, K. (1962) Zum Mechanismus der Glykolysehemmung in Ascitestumorzellen durch Röntgenstrahlen. *Strahlentherapie*, **119**, 419.

DOSE, K. & DOSE, U. (1962) The mechanism of glycolysis inhibition by X-rays in ascites tumour. *Int. J. Radiat. Biol.*, **4**, 85.

FLEMMING, K., MEHRISCH, J. N. & NAPIER, J. A. F. (1968) The loss of intracellular $K^+$ ions from the intact Ehrlich ascites carcinoma cell following irradiation. *Int. J. Radiat. Biol.*, **14**, 175.

GOLDBERG, D. M. (1971) Alkaline ribonuclease activity in response to therapeutic radiation in the human female. In *Biochemical Indicators of Radiation Injury in Man.* Vienna: International Atomic Energy Agency, p. 259

GOLDBERG, D. M., AYRE, H. A. & PITTS, J. F. (1967) Effect of radium treatment on activity and distribution of some enzymes in cancers of the human cervix uteri. *Cancer*, **20**, 1388.

GOLDBERG, D. M., WATTS, C. & HART, D. M. (1969) Effect of radium treatment on the enzyme content of vaginal fluid in cervical cancer. *Am. J. Obstet. Gynecol.*, **105**, 1192.

HILZ, H. & BERNDT, H. (1964) Die Schutzwirkung des Nicotinsaüreamid auf die Strahlenbedingte Schädigung der DNS-Synthese als Zellpopulationsproblem. *Z. Krebsforsch.*, **66**, 155.

IBSEN, K. H. (1977) Interrelationships and functions of the pyruvate kinase isoenzymes and their variant forms: a review. *Cancer Res.*, **37**, 341.

MARIE, J., GARREAU, H. & KAHN, A. (1977) Evidence for a postsynthetic proteolytic transformation of human erythrocyte pyruvate kinase into L-type enzyme. *FEBS Lett.*, **78**, 91.

MARSHALL, M. J., GOLDBERG, D. M., NEAL, F. E. & MILLAR, D. R. (1978*a*) Enzymes of glucose metabolism in carcinoma of the cervix and endometrium of the human uterus. *Br. J. Cancer*, **37**, 990.

MARSHALL, M. J., GOLDBERG, D. M., NEAL, F. E. & MILLAR, D. R. (1978*b*) Properties of glycolytic and related enzymes of normal and malignant human uterine tissues studied to optimise assay conditions. *Enzyme* (in press).

ONTKO, J. A. & MOOREHEAD (1964) Increased endogenous respiration of ascites tumour cells after radiation exposure. *Radiat. Res.*, **23**, 135.